# Benchmark-grounded self-supervised contrastive learning for AMI-based distribution grid monitoring

**DOI:** 10.3389/frai.2026.1868885

**Published:** 2026-08-31

**Authors:** Ling Zhang, Bowei Yang, Xingsi Ke, Hong Zhao, Wenhua Zhang, Yumin Yao

**Affiliations:** 1 State Grid Sichuan Electric Power Corporation, Chengdu, China; 2 School of Computer Science and Engineering, Central South University, Changsha, China

**Keywords:** cyber-physical systems, domain adaptation, lightweight neural networks, self-supervised contrastive learning, structural evolution, time-series representation learning

## Abstract

**Introduction:**

Self-supervised contrastive learning is effective for time-series representation learning, but its deployment in industrial environments remains challenging because of spatiotemporal heterogeneity, domain-specific noise, and label scarcity. We study these challenges in the smart-grid domain, a representative cyber-physical system characterized by non-stationary events, missing data, and policy-driven distribution shifts.

**Methods:**

We introduce the West China AMI Benchmark (WCA-Bench), a real-world dataset containing 713,700 user-day electricity profiles from 1,950 users and 506 expert-verified structural evolution events, including electric-vehicle access and photovoltaic installation. We further propose Domain-Aware Self-Supervised Contrastive Learning (DA-SSCL), which combines a lightweight PatchMLP encoder, physically grounded augmentations, and a hierarchical instance- and patch-level InfoNCE objective.

**Results:**

DA-SSCL achieves an unsupervised clustering ARI of 0.699, reaches 88.4% classification accuracy with only 10% labeled data, and maintains transferability across the evaluated heterogeneous city districts.

**Discussion:**

These results provide a practical methodology and a verified benchmark for robust, label-efficient, and transferable time-series representation learning in cyber-physical systems.

## 1 Introduction

Time-series representation learning is a fundamental challenge in artificial intelligence, critical for extracting actionable insights from the complex, high-dimensional temporal data generated by cyber-physical systems (Jing and Tian, 2020). While deep supervised learning has achieved remarkable success, its reliance on large-scale, high-quality annotated data creates a significant bottleneck. This label scarcity is particularly severe in specialized industrial domains where expert annotation is prohibitively expensive, privacy-restricted, or simply unavailable (Huber et al., 2021; Ryu et al., 2017; Omitaomu and Niu, 2021). Similar pressures have also been observed in other high-stakes sensing domains, where recent deep models increasingly combine transfer learning, task-specific inductive bias, and efficiency-aware design to cope with heterogeneous and annotation-scarce data (Mahmood et al., 2021, 2022, 2023, 2024a,b, 2025; Rehman et al., 2025). Consequently, Self-Supervised Learning (SSL)—and specifically Contrastive Learning—has emerged as a transformative paradigm. By designing pretext tasks that leverage the intrinsic structure of unlabeled data, contrastive methods learn generalized representations capable of supporting diverse downstream applications (Chen et al., 2020). However, current time-series SSL models are predominantly evaluated on relatively static, curated, or simplified synthetic datasets. This reliance on idealized benchmarks limits the AI community's ability to assess how these models handle the non-stationary dynamics, heterogeneous noise, and severe distribution shifts inherent in real-world temporal environments.

To evaluate representation learning methods in realistic, uncontrolled settings, there is a pressing need for practical, domain-specific testbeds. The modern smart grid, empowered by Advanced Metering Infrastructure (AMI), serves as an ideal cyber-physical ecosystem for this purpose (Seo and Huh, 2022). High-resolution electricity consumption profiles are not only massive in scale but also structurally complex: they exhibit strong periodicities heavily entangled with abrupt structural evolutions, such as the uncoordinated charging of Electric Vehicles (EVs) or the integration of distributed Photovoltaic (PV) systems (Hashmi et al., 2024). Furthermore, real-world utility data is intrinsically noisy, suffering from communication-induced missing intervals and policy-driven distribution shifts (e.g., Time-of-Use pricing). Unfortunately, there is currently a lack of open, expert-verified benchmarks that capture these rich spatiotemporal phenomena. Most existing AMI datasets are either heavily aggregated, stripped of crucial transient events, or lack the ground-truth annotations necessary to rigorously evaluate representation quality across unsupervised, semi-supervised, and cross-domain transfer settings.

Furthermore, directly transferring general self-supervised learning frameworks (such as SimCLR or MoCo) to cyber-physical time-series data remains fundamentally problematic. Common time-series augmentations—for example, random jitter, unconstrained scaling, or temporal permutation—are rarely designed around the physical constraints of real-world sensing systems. Transformations that are harmless in domains like human activity recognition or audio processing may distort physical causality, violate energy conservation laws, or erase crucial temporal signatures associated with external policy shifts, sensor missingness, and structural evolution. As a result, domain-agnostic augmentations can encourage invariances that are convenient for optimization but entirely misaligned with the underlying generative factors of the target domain.

To address this gap, we rethink the representation learning objective by grounding it in real-world physical phenomena rather than generic augmentation heuristics. We first construct the West China AMI Benchmark (WCA-Bench), an anonymized utility dataset with pronounced district-level heterogeneity and 506 verified structural evolution events. We then develop Domain-Aware Self-Supervised Contrastive Learning (DA-SSCL), an AI framework in which observed real-world sensing phenomena directly guide both the augmentation design and the invariances imposed during representation learning. Downstream clustering, cross-domain transfer, and low-label classification are subsequently used to rigorously test whether these benchmark-grounded invariances translate into measurable representation quality, transferability, and feature space separability.

The main contributions of this paper to the AI and time-series representation learning communities are as follows:

- A challenging real-world time-series benchmark: We curate and open-source the WCA-Bench, comprising 713,700 user-day profiles from 1,950 users across three heterogeneous districts. With 506 expert-verified structural evolution events, it provides a benchmark for non-stationary time-series representation learning.
- A domain-aware contrastive learning framework: We propose DA-SSCL, a time-series SSL framework that operationalizes domain awareness through physically-grounded augmentations (encoding missingness, policy-driven redistribution, and structural evolution) coupled with a hierarchical contrastive objective and a lightweight PatchMLP backbone.
- Demonstrated generalizability and transferability: Extensive experiments validate that DA-SSCL learns highly discriminative representations, achieving competitive unsupervised clustering (ARI = 0.699) and 88.4% semi-supervised classification accuracy with only 10% labels. Furthermore, we demonstrate strong cross-district transferability under the evaluated spatial distribution shifts.

## 2 Related work

### 2.1 Smart grid datasets and time-series benchmarks

Public datasets have advanced time-series analytics in cyber-physical systems. In the energy domain, household-level datasets (Pereira et al., 2022) and high-frequency measurements support non-intrusive load monitoring and appliance-level forecasting, while aggregated datasets (Hashmi et al., 2024; Quesada et al., 2024) facilitate regional demand analysis. Nevertheless, most existing resources are either highly aggregated or lack expert-verified annotations for structural temporal evolution (such as newly integrated devices or abrupt behavioral shifts). This absence limits their utility for evaluating representation learning algorithms under realistic, non-stationary operating conditions. Our WCA-Bench is explicitly designed to provide ground-truth annotations for these complex structural shifts.

### 2.2 Time-series representation learning

Extracting meaningful representations from complex time-series data traditionally relied on clustering algorithms to group similar temporal behaviors. However, these methods have limited capabilities in capturing high-dimensional and nonlinear temporal structures. In contrast, deep representation learning combines feature extraction with downstream objectives to improve performance. Recently, advanced architectures such as Transformers (Tuli et al., 2022; Zhou et al., 2022), Graph Neural Networks (GNN) (Deng and Hooi, 2021), and hybrid unsupervised frameworks (Audibert et al., 2020; Xu et al., 2024) have been proposed to enhance time-series modeling capabilities. A similar trend is also visible in other complex sensing domains, where deep learning systems increasingly rely on transfer learning, active learning, multi-modal feature fusion, and robustness-oriented representation design to improve performance under data heterogeneity and limited supervision (Mahmood et al., 2020, 2023, 2024a,b, 2025; Rehman et al., 2025). While effective, these large-scale models often incur high computational costs, motivating the need for more lightweight, representation-focused architectures like the PatchMLP used in our framework.

### 2.3 Self-supervised learning paradigms

Self-supervised learning has emerged as a primary paradigm for time-series analysis, categorized broadly into generative and contrastive approaches.

#### 2.3.1 Generative self-supervised learning

Generative SSL involves learning by reconstructing masked, corrupted, or future segments of a time series. In sensor data applications, frameworks like S2p (Chen et al., 2022) predict signals by reconstructing masked segments. While this approach preserves local waveform fidelity, it can over-reconstruct low-level noise at the expense of high-level semantic representation. Although current time-series foundation models (Ansari et al., 2024; Das et al., 2024) and forecasting models (Ferdaus et al., 2025) have expanded generative capabilities, their massive parameter counts and computational demands remain prohibitive for lightweight, edge-oriented deployment.

#### 2.3.2 Contrastive self-supervised learning

Contrastive SSL learns representations by pulling positive pairs together while pushing negative pairs apart in the embedding space. Following the success of instance-discrimination methods like SimCLR (Chen et al., 2020) in computer vision, time-series adaptations have rapidly evolved. Methods such as TS2Vec (Yue et al., 2022) and TNC (Tonekaboni et al., 2021) introduced hierarchical contrastive learning and temporal neighborhood coding. TS-TCC (Eldele et al., 2021) employs temporal and contextual contrastive learning to achieve robust classification. Further advancements incorporate frequency-aware mechanisms like TF-C (Zhang et al., 2022) and CoST (Woo et al., 2022), or multi-view perturbation strategies (Tang et al., 2024; Niu et al., 2023) to capture complex load-aware features. Learned masking and adaptive knowledge-guided schemes have also been explored (Lee et al., 2024; Li et al., 2024). Despite these advances, as Sabiri et al. (2024) emphasize, augmentation design remains the critical bottleneck. Most existing methods rely on domain-agnostic augmentations that fail to model the specific physical phenomena (e.g., policy-driven redistribution or structural evolution) dominating real-world cyber-physical data.

### 2.4 Domain-aware data augmentation

Data augmentation is fundamental to contrastive learning, dictating the invariances the model will learn. Common time-series augmentation methods include jittering, permutation, temporal regularization (Iwana and Uchida, 2021), and synthetic generation (Forestier et al., 2017). While highly effective in domains like human activity recognition where variation is inherently stochastic, they present severe challenges in cyber-physical systems. In such domains, the defined “invariance” must not violate underlying physical laws. More broadly, recent deep learning systems in safety-critical sensing have also moved toward task-aligned transformations, modality-aware fusion, and data-efficient adaptation rather than relying on purely generic perturbation pipelines (Mahmood et al., 2021, 2022, 2024a,b, 2025; Rehman et al., 2025). For instance, Stomps et al. (2024) demonstrated the need for label-preserving augmentations, noting that random permutations disrupt causal temporal structures, while unconstrained scaling violates physical capacity limits. Building on this principle, our DA-SSCL framework operationalizes domain awareness by deriving its augmentation library directly from the empirical sensing phenomena documented in the WCA-Bench.

## 3 The WCA-Bench: a real-world AMI sensing benchmark

The proposed framework is evaluated on the West China AMI Benchmark (WCA-Bench), a real-world AMI benchmark collected, processed, and validated with utility support in western China. The benchmark is designed not only as a data source but also as the empirical basis for augmentation design and downstream validation. In other words, the same sensing phenomena observed in the dataset are used to define the invariances that DA-SSCL is encouraged to learn.

### 3.1 Dataset composition and statistics

The dataset in this paper comprises 1,950 users and 713,700 daily load curves, recording 506 verified structural evolution events, including electric vehicle connections, PV installations, and sudden operational disturbances. Events were verified against utility work orders and field inspection records. The scale and district diversity reflect realistic behavioral drift, demand-response effects, and heterogeneous consumption patterns in distribution-grid monitoring. User identifiers are fully anonymized, while utility metadata—tariff contracts and user categories—are retained to support downstream evaluation. Table 1 summarizes the district composition and monitoring relevance.

**Table 1:** District composition and cross-domain characteristics of the WCA-Bench.

Sub-dataset	Dominant composition	Count	Monitoring relevance
City A	Mixed residential/commercial	800	Mixed routines, TOU-sensitive behavior
City B	Commercial-dominated district	500	Business-hour regularity, highest TOU response
City C	Mixed-use district with EV/PV changes	650	Structural evolution and cross-district transfer
**Total**	**Multi-district AMI sensing network**	**1,950**	**Heterogeneous monitoring benchmark**

Bold text indicates the aggregate summary across all three city subsets.

To improve transparency of the released benchmark, we further summarize city-level profile counts, event counts, sparsity-related zero-value ratios, and event-alignment statistics in Table 2. City A, City B, and City C contribute 292,800, 183,000, and 237,900 daily profiles, respectively, together covering the full 2024 calendar year. Because the released daily curves are min–max normalized and do not preserve an explicit binary missingness mask, we report zero-value ratios as a descriptive sparsity proxy rather than a literal missing-data label. In the curated release, the user-level event metadata and the event registry are exactly aligned on *user_id, event_type*, and *start_day*; the *start_day* field is the day-index key used to align each verified event with the corresponding AMI measurement timeline. This yields 506 internally consistent structural-evolution records in total.

**Table 2:** Additional city-level statistics of WCA-Bench.

City	Users	Daily profiles	Events	Event alignment	Zero-value ratio	Mean peak hour
City A	800	292,800	267	100.0%	16.36%	15.44
City B	500	183,000	72	100.0%	14.23%	12.95
City C	650	237,900	167	100.0%	16.11%	14.50
**Total**	**1,950**	**713,700**	**506**	**100.0%**	**15.54%**	**14.46**

Bold text indicates the aggregate summary across all three city subsets.

These statistics also make the benchmark imbalance explicit. City A, City B, and City C account for 41.0%, 25.6%, and 33.3% of the daily profiles, respectively, while their event shares are 52.8%, 14.2%, and 33.0%. This mismatch between profile share and event share reflects the imbalanced monitoring distribution faced by downstream models. The zero-value ratios differ across cities (14.23%–16.36%), indicating heterogeneous sparsity or communication-drop patterns. Seasonal variation is summarized at the shape level by the city-dependent mean peak hour, which ranges from 12.95 to 15.44 across the full-year records. Fine-grained class identifiers are retained in the anonymized release metadata for reproducible group-wise evaluation, while the manuscript reports Macro-F1 alongside accuracy to reduce the effect of class imbalance in low-label classification.

### 3.2 Significance for grid monitoring

Unlike most smart-meter datasets oriented toward residential clustering, this benchmark preserves operational events and physically meaningful variations. The documented EV, PV, and shock events provide an empirical basis for evaluating structural load evolution in distribution grids. These characteristics also directly inform the design of DA-SSCL: missing intervals motivate temporal masking, TOU-sensitive shifts motivate peak-shifting views, district heterogeneity motivates transferable embeddings, and verified structural events require the encoder to remain sensitive to meaningful load evolution rather than static user categories alone. Consequently, the benchmark serves not only as an evaluation corpus but also as the empirical basis for defining the invariances and sensitivities that the representation model should preserve.

### 3.3 Comparison with existing datasets

Existing smart-meter datasets have different focuses, but only a limited number support the study of structural load evolution in distribution grids. Household-level datasets such as SustDataED2 (Pereira et al., 2022) are primarily used for appliance-level analysis and non-intrusive load monitoring. Datasets focusing on photovoltaic generation or aggregated electric vehicle charging (Hashmi et al., 2024) describe specific grid-side phenomena; however, their application scope remains relatively narrow. A large regional dataset of Spanish households (Quesada et al., 2024) is suitable for forecasting and price-response studies at scale, but lacks verified event annotations for operational changes.

By contrast, the WCA-Bench is explicitly designed for monitoring analysis in active distribution grids. It contains daily AMI records of 1,950 users together with 506 verified EV, PV, and shock events. Consequently, changes in load shapes can be analyzed alongside their practical operational context. This enables the dataset to be used not only for general representation learning evaluation, but also for verifying whether the learned features capture meaningful operational variations. These benchmark characteristics directly motivate the methodology presented in the next section, where observed AMI phenomena are converted into augmentation rules and representation constraints for DA-SSCL.

## 4 Methodology

### 4.1 Overview

**Figure 1:**
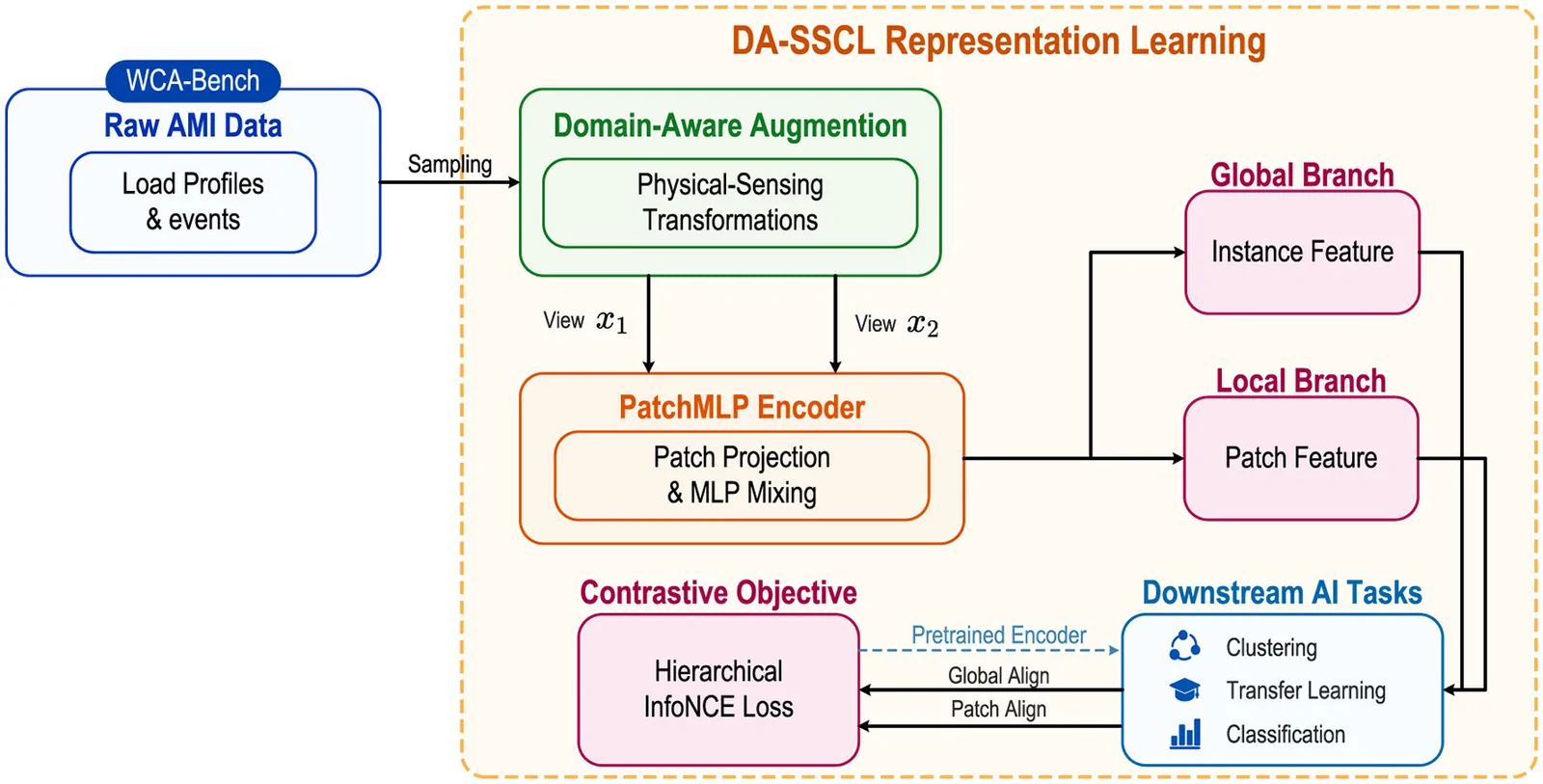
Overview of the proposed DA-SSCL framework. Illustrating the flow from raw time-series data through domain-specific augmentations to the hierarchical contrastive learning objective. The input sequence is divided into localized patches and processed by a lightweight PatchMLP encoder. The optimization aligns both the global instance-level representations and the fine-grained patch-level features across augmented views. This benchmark-driven design forms the foundation of the AI pipeline, ensuring that the learned invariances directly address spatiotemporal heterogeneity and physical sensing constraints.

Based on the benchmark characteristics summarized in Section 3, DA-SSCL converts observed AMI phenomena into explicit learning constraints. The framework consists of three coupled modules: a domain-specific augmentation bank, a lightweight PatchMLP encoder, and a hierarchical contrastive objective. The augmentation bank produces positive views using only benchmark-supported perturbations, such as communication missingness, TOU-driven reshaping, and season-sensitive modulation. The encoder maps each daily curve to a compact global embedding and a set of patch-level features. The optimization objective then aligns the global and local representations so that the learned embedding remains invariant to admissible AMI disturbances while preserving discriminative information for user profiling and structural-evolution screening. This benchmark-to-model mapping is the principal distinction between DA-SSCL and generic time-series SSL pipelines. An overview of the complete DA-SSCL framework is presented in Figure 1.

### 4.2 Domain-specific data augmentation

#### 4.2.1 Design principles

The augmentation module is the main component that distinguishes DA-SSCL from generic contrastive learning. Standard SSL pipelines usually generate positive views through generic perturbations that may aid optimization but do not necessarily preserve monitoring-relevant semantics. In DA-SSCL, positive pairs are generated only from perturbations corresponding to recurring AMI sensing phenomena verified in the benchmark. The learned invariances are therefore tied to realistic changes in smart-meter data rather than heuristic augmentation choices.

Unlike generic augmentations for images or unconstrained time-series data, the augmentation bank is organized around three monitoring objectives: robustness to AMI sensing failures and noise, invariance to TOU-driven load shifting and demand-response behavior, and tolerance to seasonal or weather-related variation. Some operations, such as temporal masking and peak shifting, can be viewed as grid-constrained variants of common time-series transformations (Le Guennec et al., 2016). However, they are retained only when they remain physically reasonable, energy-consistent when required, and non-destructive to monitoring-related information.

#### 4.2.2 Benchmark-to-augmentation mapping

The mapping from benchmark phenomena to augmentations is designed in a one-to-one manner. Missing intervals and communication drops motivate *Temporal masking*; routine metering fluctuation motivates *Periodic perturbation*; TOU-sensitive peak avoidance motivates *Peak shifting* and *Load redistribution*; and climate- or season-induced demand variability motivates *Seasonal modulation* and *Weather impact*. For each transformation, the parameter range is constrained by empirical AMI behavior so that robustness is improved without disrupting monitoring-related semantics.

#### 4.2.3 View composition

In implementation, each positive view is generated by composing a small number of admissible perturbations rather than applying a single fixed operator. Let $A=\left\{a_{1},\ldots ,a_{6}\right\}$ denote the augmentation bank. For an input curve *x*, we sample *u*~*U*{1, 2, 3} operators without replacement and construct the augmented view as shown in Equation 1


$$\begin{array}{l} \tilde{x}=a_{k_{u}}○\cdots ○a_{k_{2}}○a_{k_{1}}\left(x\right), \end{array}$$ (1)

followed by clipping to the physically meaningful normalized range [0, 1]. This stochastic composition enlarges the positive-view space while keeping each view within the operating envelope observed in the benchmark.

#### 4.2.4 Mathematical formulations

Table 3 summarizes the augmentation library. Each transformation expands the view space while retaining a clear interpretation with respect to user state, operational context, and structural identity. In this way, augmentation is treated not as generic regularization, but as a direct mechanism for encoding benchmark-grounded invariances into feature learning.

**Table 3:** Benchmark-grounded domain-specific augmentation bank for time-series representation learning.

Augmentation strategy	Description and formulation
Temporal masking (Data quality)	Simulates AMI missingness caused by communication outages or sensor drops while preserving the underlying daily behavior. It zeros out a segment of length *l* starting at *s*: $X_{\text{masked}}^{\left(t\right)}=\{\begin{array}{ll} 0 & s\leq t<s+l\\ X^{\left(t\right)} & \text{otherwise} \end{array}$
Periodic perturbation (Data quality)	Models low-amplitude sensing disturbance and routine drift by injecting noise proportional to the signal magnitude: $X_{\text{perturbed}}^{\left(t\right)}=X^{\left(t\right)}\cdot (1+\epsilon_{t}),\;\epsilon_{t}\sim N(0,\sigma^{2})$
Peak shifting (Demand response)	Simulates TOU-driven peak avoidance or DR scheduling, enforcing invariance to feasible temporal displacement of high-load activity: $X_{\text{shifted}}^{\left(t\right)}=X^{\left(t+\delta \right)}\text{ for }t\in T_{\text{peak}}\text{ with prob. }p_{\text{shift}}$
Load redistribution (Demand response)	Reflects TOU-based load transfer by moving a portion β of peak load to valley periods while conserving total energy: $X_{\text{new}}^{\left(t\right)}=\{\begin{array}{ll} (1-\beta )X^{\left(t\right)} & t\in T_{\text{peak}}\\ X^{\left(t\right)}+\Delta_{\text{valley}} & t\in T_{\text{valley}} \end{array}$ where $\Delta_{\text{valley}}=\frac{\beta \underset{t\in T_{\text{peak}}}{\sum }X^{\left(t\right)}}{\left|T_{\text{valley}}\right|}$ ensures energy conservation.
Seasonal modulation (External factors)	Captures smooth seasonal or calendar-driven variation without changing the essential load-profile structure: $X_{\text{seasonal}}^{\left(t\right)}=X^{\left(t\right)}\cdot \left[1+\alpha \sin\left(\frac{2\pi t}{P_{\text{season}}}\right)\right]$
Weather impact (External factors)	Simulates climate-sensitive amplification or suppression under heat or cold waves using a random factor γ_*t*_: $X_{\text{weather}}^{\left(t\right)}=X^{\left(t\right)}\cdot \max(0,1+\gamma_{t}),\;\gamma_{t}\sim N(0,\sigma_{\text{weather}}^{2})$

#### 4.2.5 Bias control and scope

Because some transformations are guided by domain knowledge, DA-SSCL also requires explicit safeguards against augmentation bias. We therefore constrain every operator to empirically observed parameter ranges, limit each view to 1–3 composed operations, preserve energy when physical conservation is required, and clip the final output to the normalized operating range. In addition, the leave-one-out ablation in Section 5.3 shows that the contribution of individual operators is heterogeneous rather than uniformly positive, which helps avoid overstating the role of any single handcrafted prior. The current augmentation bank should therefore be interpreted as a benchmark-supported invariance approximation rather than a complete description of all future grid behaviors.

### 4.3 Temporal feature encoder

To capture both local variations and global daily patterns while maintaining computational efficiency, we adopt a Patch-Independent Multi-Layer Perceptron (PatchMLP) encoder. Unlike temporal convolutional networks or self-attention mechanisms, PatchMLP processes non-overlapping load-curve segments with linear complexity O(L), making it suitable for utility-side deployment scenarios with limited computing resources. In the default configuration used in this paper, a 24-point daily curve is split into six non-overlapping patches with patch size *p* = 4, and each patch is projected to a 32-dimensional token.

Consider a one-dimensional daily load curve *x* ∈ ℝ^*L*^, which is divided into *P* = *L*/*p* non-overlapping patches, where *p* is the patch size. Each patch is then mapped to a *D*_*model*_-dimensional token, as defined in Equation 2.


$$\begin{array}{l} Z^{\left(0\right)}=\text{PatchProj}\left(x\right)\in {\mathbb{R}}^{P\times D_{model}} \end{array}$$ (2)

The encoder then applies lightweight residual MLP blocks that alternate between time mixing and feature mixing, as defined in Equations 3 and 4, respectively. Time mixing enables each patch to access global daily information, which is important for capturing seasonal patterns and TOU-driven load shifts, while feature mixing preserves the patch-wise processing of local consumption details:


$$\begin{array}{l} Z^{\left(1\right)}=Z^{\left(0\right)}+\text{TimeMix}{\left({\left(Z^{\left(0\right)}\right)}^{T}\right)}^{T} \end{array}$$ (3)

$$\begin{array}{l} Z^{\left(2\right)}=Z^{\left(1\right)}+\text{FeatureMix}\left(Z^{\left(1\right)}\right) \end{array}$$ (4)

After two such blocks, the token sequence is used in two branches. A patch head produces normalized patch features for local contrastive matching, while a compact projection head flattens the tokens and outputs a normalized 16-dimensional embedding *z* ∈ ℝ^16^ for instance-level learning. This design is deliberately lightweight: the full encoder contains about 15k trainable parameters, yet still provides sufficient capacity to model daily load semantics. Compared with heavier TCN-, Transformer-, or state-space-based alternatives, it offers a more favorable trade-off between representation quality and deployment cost. This choice is motivated by the benchmark regime considered here: the input horizon is short (*L* = 24), the dominant challenge lies more in learning robust invariances than in modeling ultra-long temporal memory, and utility deployment benefits more directly from low latency and small memory footprint than from higher-capacity sequence backbones.

### 4.4 Hierarchical contrastive optimization

Based on the augmentation design in Section 4.2, DA-SSCL adopts a hierarchical contrastive objective to capture both whole-day load semantics and local consumption patterns at two levels: the instance level (entire daily profile) and the patch level (local temporal segments). The two losses are optimized jointly so that both global user structure and local temporal signatures are preserved under benchmark-grounded perturbations.

At the instance level, an InfoNCE loss is used to optimize the global embedding *z* ∈ ℝ^16^ by maximizing the consistency between an anchor and its positive view, while minimizing the similarity to other samples. For an anchor embedding *z*_*i*_, let *z*_*j*(*i*)_ denote its positive view. The negative pool combines in-batch negatives and queue-based historical negatives:


$$\begin{array}{l} N_{i}=\left\{z_{\ell }:\ell \neq i,\;\ell \neq j\left(i\right)\right\}\cup Q, \end{array}$$ (5)

where Q stores detached embeddings from recent mini-batches. The positive sample *z*_*j*(*i*)_ is included in the InfoNCE denominator only through the unweighted positive term, while hard-negative weights are applied only to samples in $N_{i}$.

We further introduce hard-negative mining (Robinson et al., 2021), because users exposed to similar tariffs or schedules may exhibit superficially similar daily curves. The weighting function emphasizes nearby but still distinct negatives in the feature space:


$$\begin{array}{l} \omega_{ik}=\frac{\exp\left(\beta \cdot \text{sim}\left(z_{i},z_{k}\right)\right)}{\sum_{m\in N_{i}}\exp\left(\beta \cdot \text{sim}\left(z_{i},z_{m}\right)\right)},\;k\in N_{i}, \end{array}$$ (6)

where β denotes the control parameter for hard-negative emphasis. The resulting instance-level objective is


$$\begin{array}{l} L_{\text{inst}}=-\log\frac{\exp\left(\text{sim}\left(z_{i},z_{j\left(i\right)}\right)/\tau \right)}{\exp\left(\text{sim}\left(z_{i},z_{j\left(i\right)}\right)/\tau \right)+\sum_{k\in N_{i}}\omega_{ik}\exp\left(\text{sim}\left(z_{i},z_{k}\right)/\tau \right)}. \end{array}$$ (7)

This formulation makes three implementation details explicit. First, the positive view is not treated as a negative. Second, hard-negative weights are applied only to in-batch and queue-based negatives. Third, the queue is concatenated with the current in-batch negative set before computing ω_*ik*_, and it is updated only after the current optimization step. The method is therefore ranking-based rather than threshold-based: negatives with larger cosine similarity to the anchor automatically receive higher weights under Equation 6, while dissimilar negatives remain in the denominator with smaller weights.

At the patch level, the model enforces consistency between corresponding temporal segments in the two augmented views. For patch features *p*_*i, m*_ and *p*_*j, m*_, a local contrastive loss $L_{\text{patch}}$ is computed across all *M* patches and then averaged. This term discourages the encoder from preserving only the global daily shape while ignoring discriminative local shifts such as peak relocation or daytime suppression. The final objective combines the global and local consistency:


$$\begin{array}{l} L_{\text{total}}=L_{\text{inst}}+\alpha L_{\text{patch}} \end{array}$$ (8)

A queue-based memory bank is used to maintain negative samples beyond the current mini-batch. In addition, the temperature coefficient τ is treated as a learnable parameter so that adaptive similarity scaling can be achieved during training. In this way, hierarchical contrastive learning, hard-negative weighting, memory-augmented negatives, and adaptive temperature control jointly improve representation quality for monitoring-oriented tasks. For cross-city transfer, the same objective is first optimized on the source city and then continued on unlabeled target-city data before prototype-based evaluation with a small labeled support set.

### 4.5 Algorithm summary

Algorithm 1 summarizes the complete training procedure of DA-SSCL.

Algorithm 1 Domain-aware self-supervised contrastive learning (DA-SSCL). 
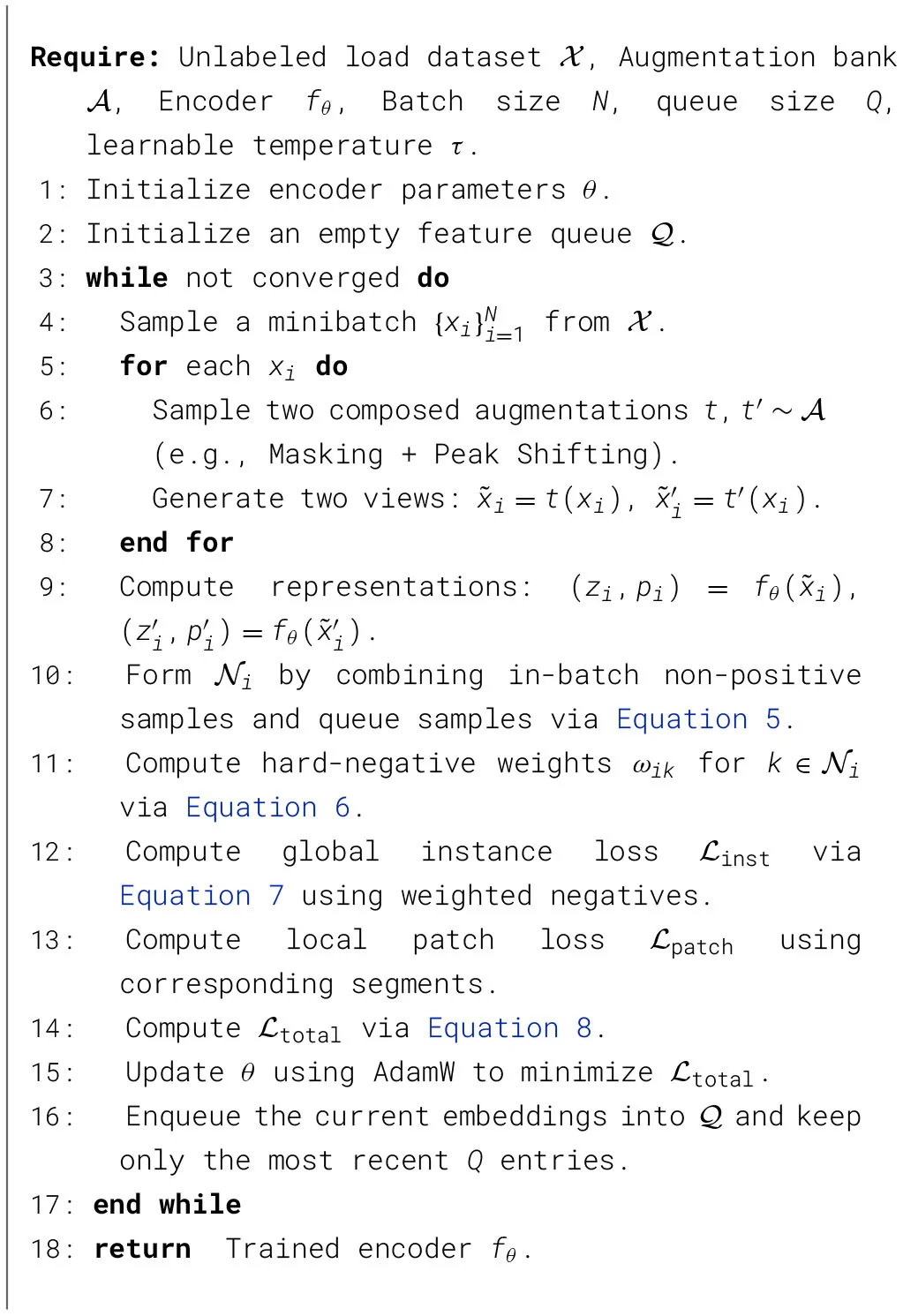

### 4.6 Comparison with representative self-supervised learning baselines

DA-SSCL has a different position from the baselines considered in this study. Compared with reconstruction-based methods such as S2p (Chen et al., 2022), it learns invariances from benchmark-grounded perturbations instead of waveform restoration. Compared with generic contrastive methods such as TS2Vec (Yue et al., 2022), Series2Vec (Foumani et al., 2024), and DCdetector (Niu et al., 2023), it limits the invariant space to AMI transformations supported by the benchmark. Different from larger foundation-style backbones such as MOMENT (Goswami et al., 2024), DA-SSCL combines a compact PatchMLP encoder with monitoring-oriented contrastive objectives. The framework is applied to daily and weekly AMI sequences (*L* = 24 and *L* = 168), and the encoder complexity scales linearly with sequence length, O(L), as summarized in Table 4.

**Table 4:** Coarse complexity classes of representative baselines.

Model	Backbone	Complexity class	Params
MOMENT16 (Goswami et al., 2024)	Patch transformer	Attention-dominant	73k
TSDE16 (Senane et al., 2024)	Diffusion + transformer	Diffusion-enhanced	21k
DCdetector (Niu et al., 2023)	Dual attention	Attention-dominant	45k
Series2Vec16 (Foumani et al., 2024)	Convolutional encoder	Convolution-dominant	12k
**DA-SSCL_Patch16**	**PatchMLP**	**Linear/MLP-dominant**	**15k**

Bold text identifies the proposed DA-SSCL_Patch16 model and its specifications.

### 4.7 Theoretical justification

DA-SSCL can be understood as a benchmark-grounded consistency learning framework. Its objective is not to reconstruct every local waveform detail, but to preserve the information that remains stable under admissible AMI perturbations while suppressing nuisance variation caused by sensing noise, tariff reshaping, or seasonal fluctuation. This objective is consistent with the Information Bottleneck view of representation learning, in which the encoder should retain monitoring-relevant user identity and structural state while discarding factors that are not useful for profiling or event screening.

The InfoNCE objective provides the formal basis for this design through the mutual-information lower bound shown in Equation 9:


$$\begin{array}{l} I\left(Z_{i};Z_{j}\right)\geq \log\left(N\right)-L_{\text{inst}} \end{array}$$ (9)

where *N* is the number of negative samples. Let an observed load curve be decomposed conceptually as *x* = *g*(*s, n*), where *s* denotes monitoring-relevant latent factors such as user behavior or structural state, and *n* denotes nuisance variation induced by metering noise, seasonal fluctuation, or policy-driven reshaping. DA-SSCL restricts its positive transformations to a benchmark-supported set A such that, ideally, *a*(*x*) = *g*(*s, n*′) for a∈A, meaning that admissible augmentations change nuisance factors while preserving the underlying state *s*. Under this assumption, minimizing $L_{\text{inst}}$ increases agreement between views that share the same operational semantics, while the patch-level loss $L_{\text{patch}}$ prevents fine-grained local signatures such as peak timing and localized suppression from being over-smoothed by global averaging. Following the alignment–uniformity perspective of Wang and Isola (2020), hard-negative mining further separates users with superficially similar but semantically different patterns. In this sense, the training objective can be interpreted as maximizing information about *s* while suppressing sensitivity to nuisance factors *n*, which provides a more concrete information-bottleneck interpretation of benchmark-supported invariance learning.

## 5 Results

### 5.1 Experimental setup

#### 5.1.1 Datasets and evaluation protocol

All experiments are conducted on WCA-Bench. Because the benchmark contains verified structural events, district heterogeneity, and TOU-sensitive behavior, it provides a direct test of the proposed operational validation framework: if augmentations are correctly derived from observed AMI phenomena, they should improve representation quality beyond generic SSL recipes. To avoid information leakage, all data splits are enforced at the user level. For the mixed *AllCities* benchmark, users are divided into 70%/10%/20% train/validation/test partitions. For cross-city transfer, source and target districts are kept strictly isolated: the model is pretrained on the source city, while target-city users are split into train/validation/test partitions, after which 10% of the target training users are sampled class-wise as support users and the remaining target training users are used as unlabeled adaptation data. This protocol matches the practical deployment setting in which a utility may have abundant unlabeled data in a new district but only limited labeled target supervision.

Daily curves are min–max normalized per profile to preserve shape information, and the retained utility metadata provide the monitoring-oriented reference groups used for clustering and low-label classification. Representation quality is assessed by K-means clustering; for the mixed AllCities setting, *K* = 8 is selected to match the eight monitoring reference groups present in the curated release. In transfer experiments, target-city embeddings are evaluated by both clustering quality and prototype-based classification. To isolate the contribution of each stage, we compare three transfer strategies: *source-only* (source pretraining without target adaptation), *target-only* (target self-supervision only), and *full transfer* (source pretraining followed by unlabeled target adaptation). All splits are made at the user level before training, so all daily profiles from the same user remain in the same partition. This prevents leakage in which different days from a test user appear during pretraining or classifier fitting. We nevertheless acknowledge that a purely chronological split would be needed to evaluate future-year temporal drift, which is discussed as a limitation in Section 6.3. Table 5 summarizes the metrics used throughout the study.

**Table 5:** Evaluation metrics used for clustering and low-label classification.

Metric	Definition and formulation
Adjusted rand index (ARI)	Measures the similarity between clustering results, adjusted for chance. It is defined based on the contingency table of *Y* and *C*: ARI=∑ijnij2-[∑iai2∑jbj2]/n212[∑iai2+∑jbj2]-[∑iai2∑jbj2]/n2 where *n*_*ij*_ is the count of samples in both cluster *i* and class *j*; *a*_*i*_ is the size of cluster *i*; and *b*_*j*_ is the size of class *j*.
Normalized mutual information (NMI)	A normalization of the mutual information (MI) score to quantify the dependence between distributions: $NMI\left(Y,C\right)=\frac{2\cdot I\left(Y;C\right)}{H\left(Y\right)+H\left(C\right)}$ where $I\left(Y;C\right)=\underset{y,c}{\sum }p\left(y,c\right)\log\frac{p\left(y,c\right)}{p\left(y\right)p\left(c\right)}$ is the mutual information, and *H*(·) denotes the entropy.
Silhouette coefficient (SC)	Evaluates the validity of consistency within clusters. The global score is the mean of individual coefficients: $SC=\frac{1}{N}\overset{N}{\underset{i=1}{\sum }}\frac{b\left(i\right)-a\left(i\right)}{\max\left\{a\left(i\right),b\left(i\right)\right\}}$ where *a*(*i*) is the mean intra-cluster distance for sample *i*, and *b*(*i*) is the mean nearest-cluster distance.
Accuracy (Acc)	Measures the fraction of correctly predicted downstream labels: $Acc=\frac{1}{N}\overset{N}{\underset{i=1}{\sum }}⊮\left[{ŷ}_{i}=y_{i}\right]$
Macro F1	Computes the unweighted mean of per-class F1 scores to reduce the effect of class imbalance: $Macro\text{-}F1=\frac{1}{K}\overset{K}{\underset{k=1}{\sum }}\frac{2\cdot Precision_{k}\cdot Recall_{k}}{Precision_{k}+Recall_{k}}$

*Y* and *C* denote the ground truth labels and predicted clusters, respectively.

#### 5.1.2 Implementation details

All experiments are implemented in PyTorch and executed on an NVIDIA RTX 3090 GPU. The default DA-SSCL encoder uses input length *L* = 24, patch size *p* = 4, model dimension 32, two MixMLP blocks, time-mixing hidden dimension 16, feature-mixing hidden dimension 48, and embedding dimension 16, yielding 15,293 trainable parameters. The augmentation bank follows the benchmark-derived ranges used in the implementation: masking length 2–6, perturbation noise σ = 0.06, peak-shift probability 0.8, load-redistribution ratio β ∈ [0.10, 0.30], seasonal modulation amplitude α ∈ [0.05, 0.18], weather perturbation σ_weather_ = 0.08, and 1–3 composed operations per view.

Optimization uses AdamW with learning rate 10^−3^, weight decay 10^−4^, and batch size 256. The patch-level loss weight is set to 0.5, the hard-negative weighting coefficient is 2.0, the feature queue size is 2,048, and the temperature parameter τ is initialized to 0.20 and learned dynamically during training. Early stopping with patience 3 is applied based on validation loss. For transfer adaptation, the target-stage learning rate is set to 5 × 10^−4^ and the unlabeled adaptation phase runs for four epochs. Unless otherwise stated, the reported results are averaged over three random seeds, and all baselines are evaluated under the same clustering or low-label downstream protocol for fair comparison. This setup is intended to isolate the effect of benchmark-grounded invariance design rather than changes in evaluation protocol.

To further quantify deployment cost, we additionally measure inference latency and throughput of the PatchMLP encoder under CPU inference, which is the more constrained utility-side deployment setting considered in this study. As summarized in Table 6, the model maintains sub-millisecond latency at small batch sizes and high throughput at larger batch sizes, while preserving a very small parameter budget. The measured incremental memory footprint is below 1 MB for all tested CPU batches. These results complement the parameter-count comparison in Table 7 by showing that the lightweight design also translates into low practical inference overhead.

**Table 6:** Runtime summary of the PatchMLP encoder on CPU inference.

Device	Batch	Params	Forward FLOPs	Latency (ms)	Throughput (samples/s)	Peak memory (MB)
CPU	1	15,293	125,440	0.451	2,216.7	< 1
CPU	64	15,293	8,028,160	0.823	77,731.8	< 1
CPU	256	15,293	32,112,640	1.308	195,663.6	< 1

**Table 7:** Comparison of feature representation quality on AllCities (mean ± std over three seeds).

Method	Params	ARI	NMI	SC
Raw24 + K-means	N/A	0.545 ± 0.064	0.645 ± 0.034	0.429 ± 0.014
Raw48 + K-means	N/A	0.458 ± 0.072	0.574 ± 0.046	0.266 ± 0.010
Fusion16 + K-means	N/A	0.452 ± 0.064	0.614 ± 0.029	0.163 ± 0.021
MOMENT16 (Goswami et al., 2024)	73k	0.435 ± 0.062	0.574 ± 0.043	0.412 ± 0.061
TSDE16 (Senane et al., 2024)	21k	0.461 ± 0.037	0.583 ± 0.006	0.352 ± 0.019
DCdetector (Niu et al., 2023)	45k	0.562 ± 0.082	0.655 ± 0.051	0.488 ± 0.041
Series2Vec16 (Foumani et al., 2024)	12k	0.594 ± 0.108	0.695 ± 0.062	0.517 ± 0.036
**DA-SSCL_Patch16 (ours)**	15k	**0.699** **±0.057**	**0.763** **±0.024**	**0.630** **±0.019**

Bold values indicate the best performance for each metric; the proposed method is also shown in bold for identification.

### 5.2 Performance comparison

With this common evaluation protocol fixed, we first compare DA-SSCL with representative baselines on the mixed AllCities benchmark. Table 7 gives the comparison results of DA-SSCL and several baseline methods, including direct-feature baselines (Raw24, Raw48, and Fusion16) and representative self-supervised methods such as MOMENT (Goswami et al., 2024), Series2Vec (Foumani et al., 2024), TSDE (Senane et al., 2024), and DCdetector (Niu et al., 2023). The suffix “16” in MOMENT16, TSDE16, Series2Vec16, Fusion16, and DA-SSCL_Patch16 denotes the 16-dimensional representation used for downstream K-means or prototype classification, not a separate published model variant.

Table 7 shows that DA-SSCL achieves the best results on all three clustering metrics. Compared with the strongest SSL baseline, Series2Vec16, ARI increases from 0.594 to 0.699, NMI from 0.695 to 0.763, and SC from 0.517 to 0.630. The gain over Raw24 is also substantial, with ARI improving from 0.545 to 0.699. From a monitoring perspective, higher ARI and NMI indicate better alignment between the learned embedding and the reference operational groups, while a higher silhouette score indicates a more separable embedding geometry for downstream screening and profiling tasks.

These gains are obtained with only 15k trainable parameters, which is smaller than most deep SSL baselines reported in Table 7. This suggests that the improvement is not simply a consequence of model scale, but of the invariances enforced during pretraining. Benchmark-grounded perturbation design and hierarchical alignment therefore improve representation usefulness more effectively than merely increasing backbone complexity.

### 5.3 Ablation studies

After establishing the overall advantage of DA-SSCL, we next examine which part of the design is responsible for this gain. To directly ablate DA-SSCL rather than only compare fused input features, the revised ablation focuses on the self-supervised training components themselves. We use two controls under the same AllCities protocol: a *no augmentation* setting, in which identical views are used in both branches, and a leave-one-out design, in which each domain-aware augmentation is removed once from the full augmentation bank. Table 8 reports the averaged results over three random seeds. A complementary objective-level ablation is provided in Table 9, where α = 0 corresponds to removing the patch-level contrastive loss.

**Table 8:** Augmentation ablation on the AllCities benchmark (mean± std over three seeds).

Setting	ARI	NMI	SC	Accuracy	Macro F1
No augmentation	0.3482 ± 0.0596	0.5102 ± 0.0761	0.1173 ± 0.0109	0.6615 ± 0.0644	0.5458 ± 0.1240
Drop temporal masking	0.5541 ± 0.0779	0.6696 ± 0.0624	0.2241 ± 0.0121	0.8709 ± 0.0434	0.8494 ± 0.0317
Drop periodic perturbation	0.6642 ± 0.0241	0.7674 ± 0.0287	0.3938 ± 0.0171	0.9120 ± 0.0348	0.9014 ± 0.0446
Drop peak shifting	0.5716 ± 0.0201	0.6818 ± 0.0250	0.3704 ± 0.0358	0.8530 ± 0.0359	0.8249 ± 0.0424
Drop load redistribution	0.6199 ± 0.0442	0.7281 ± 0.0361	0.3734 ± 0.0186	0.9017 ± 0.0039	0.8801 ± 0.0069
Drop seasonal modulation	0.6367 ± 0.0603	0.7584 ± 0.0361	0.3910 ± 0.0066	0.8829 ± 0.0065	0.8666 ± 0.0044
Drop weather impact	0.6687 ± 0.0274	0.7667 ± 0.0207	0.4119 ± 0.0564	0.8701 ± 0.0720	0.8328 ± 0.1180

**Table 9:** Sensitivity analysis of the patch-level loss weight α on the AllCities benchmark (mean ± std over three seeds).

α	ARI	NMI	SC	Accuracy	Macro F1
0.0	0.6348 ± 0.0415	0.7557 ± 0.0183	0.4509 ± 0.0529	0.8735 ± 0.0423	0.8342 ± 0.0754
0.1	0.6509 ± 0.0208	0.7631 ± 0.0017	0.4707 ± 0.0322	0.8538 ± 0.0179	0.8012 ± 0.0427
0.3	0.6127 ± 0.0517	0.7437 ± 0.0161	0.3745 ± 0.0328	0.8983 ± 0.0065	0.8831 ± 0.0099
0.5	0.6139 ± 0.0364	0.7248 ± 0.0265	0.3656 ± 0.0291	0.9137 ± 0.0180	0.9031 ± 0.0267
0.8	0.6443 ± 0.0336	0.7518 ± 0.0264	0.3466 ± 0.0096	0.9085 ± 0.0039	0.8951 ± 0.0058
1.0	0.6578 ± 0.0505	0.7512 ± 0.0242	0.3338 ± 0.0126	0.8966 ± 0.0233	0.8849 ± 0.0267

Table 8 first shows that the augmentation library is important as a whole. Removing all augmentations degrades every metric, reducing ARI to 0.3482 and accuracy to 0.6615. This result indicates that benchmark-grounded view generation is not an incidental implementation detail, but a central mechanism through which DA-SSCL acquires monitoring-relevant invariances.

The leave-one-out results further indicate that the contribution of individual augmentations is heterogeneous rather than uniform. Removing *Peak Shifting* or *Temporal Masking* leads to the clearest degradation, indicating that these two operators are the most influential components on the current benchmark. By contrast, the impact of removing *Load Redistribution* or *Seasonal Modulation* is moderate, while the marginal effect of *Periodic Perturbation* and *Weather Impact* is more task-dependent. We therefore revise the interpretation accordingly: the full augmentation bank is beneficial overall, but the relative importance of individual transformations differs across clustering geometry and downstream classification.

### 5.4 Transferability analysis

The ablation results explain why the method works on the mixed benchmark, but practical deployment also requires the learned representation to remain useful across districts. To evaluate whether DA-SSCL is genuinely transferable rather than city-specific, we train on the commercial-dominated City B and evaluate transfer to the mixed-use City C under three controls: *source-only, target-only*, and *full transfer*. The last setting combines source-city pretraining with unlabeled target-city adaptation and therefore tests the full DA-SSCL transfer pipeline.

Table 10 reveals a clear pattern. First, *full transfer* outperforms *source-only* on all target metrics, improving accuracy from 0.8308 to 0.8974 and Macro F1 from 0.8216 to 0.8967. This verifies that unlabeled target adaptation is not a marginal refinement but a necessary stage for correcting domain mismatch between districts. Second, *full transfer* also outperforms *target-only*, especially in clustering quality, where ARI increases from 0.6360 to 0.7260. This shows that the transferred source prior contributes information that cannot be recovered by target-city self-supervision alone.

**Table 10:** Transfer learning controls on City B → City C (mean ± std over three seeds).

Strategy	ARI	NMI	Target accuracy	Macro F1
Source-only	0.6618 ± 0.0109	0.7926 ± 0.0364	0.8308 ± 0.0353	0.8216 ± 0.0457
Target-only	0.6360 ± 0.1153	0.7764 ± 0.0653	0.8538 ± 0.0480	0.8592 ± 0.0385
**Full transfer**	**0.7260** **±0.0454**	**0.8146** **±0.0227**	**0.8974** **±0.0194**	**0.8967** **±0.0110**

Bold values indicate the best performance among the compared transfer strategies.

An additional observation is that *target-only* already attains better classification performance than *source-only*, but remains noticeably weaker and less stable in clustering. This indicates that target-city self-supervision helps adapt to local decision boundaries, yet without source-city pretraining and unlabeled adaptation the learned space remains less structured. The best transfer performance therefore emerges only when the two stages are combined: source pretraining supplies transferable behavioral structure, and target adaptation reshapes that structure to match the new district composition.

### 5.5 Hyperparameter sensitivity analysis

Following the transfer experiment, we examine two important hyperparameters: the patch-level loss weight α in Equation 8 and the fusion dimension *D*. The goal is to clarify the trade-off between local consistency and global separability while also identifying a compact embedding setting for downstream tasks.

Table 9 shows that α controls a clear trade-off between clustering geometry and downstream classification. Smaller values favor global clustering quality, with α = 0.1 achieving the best NMI and silhouette score, while larger values can improve ARI. By contrast, the best downstream classification performance is obtained at α = 0.5, which reaches 0.9137 accuracy and 0.9031 Macro F1. We therefore retain α = 0.5 as the default setting, not because it is uniformly best on every metric, but because it provides the most balanced trade-off between global separability and local discriminative consistency.

**Figure 2:**
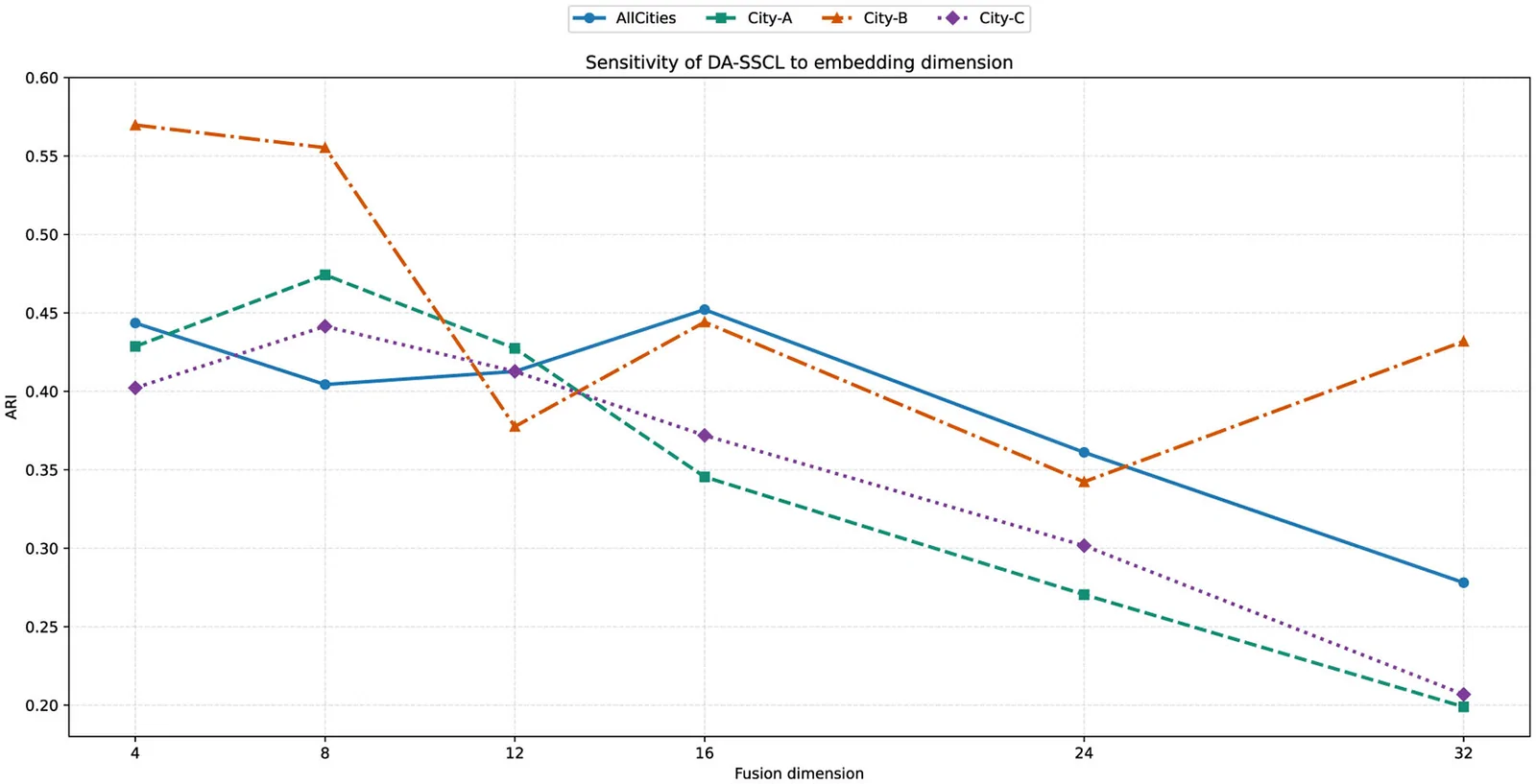
Sensitivity analysis of DA-SSCL performance (ARI) with respect to fusion dimension. The results indicate that a moderate fusion dimension is sufficient for preserving useful monitoring-related patterns.

Turning to the embedding size, Figure 2 shows that the effect of *D* is non-monotonic and differs across cities. On the AllCities benchmark, the highest ARI is obtained at *D* = 16. For the individual cities, better results are usually observed at smaller dimensions, most often around *D* = 4 or *D* = 8, which is consistent with differences in user composition and local operating patterns. When *D* increases to 24 or 32, performance drops in most cases, suggesting that a larger fusion space offers limited benefit and may introduce redundancy. We therefore use *D* = 16 in the main experiments because it provides the best balance between capacity, stability, and cross-city reuse. This choice is also consistent with the transfer control results in Table 10, where compact embeddings remain sufficient to support both clustering and low-label classification.

### 5.6 Semi-supervised classification

After identifying a balanced fusion dimension, we next ask whether the learned representation remains useful when downstream labels are scarce. This section evaluates the label efficiency through semi-supervised classification. A pretrained DA-SSCL encoder is combined with limited labeled data fractions (1%, 5%, 10%, 20%, 50%, 100%) and then assessed by a k-nearest-center classifier, in order to test whether the unsupervised representation has already captured most of the downstream class structure.

For the k-nearest-center classifier, the encoder is first frozen and all support samples are mapped to normalized embeddings. For each class *c*, the class prototype is computed from the labeled support users according to Equation 10:


$$\begin{array}{l} \mu_{c}=\frac{1}{\left|S_{c}\right|}\underset{i\in S_{c}}{\sum }z_{i}, \end{array}$$ (10)

where *S*_*c*_ denotes the support set of class *c*. A test embedding is assigned to the class with the largest cosine similarity to μ_*c*_ (equivalently, the nearest prototype on the normalized embedding space). For each label ratio and random seed, support users are sampled class-wise from the training split and prototypes are recomputed; test users are never used in prototype estimation.

**Figure 3:**
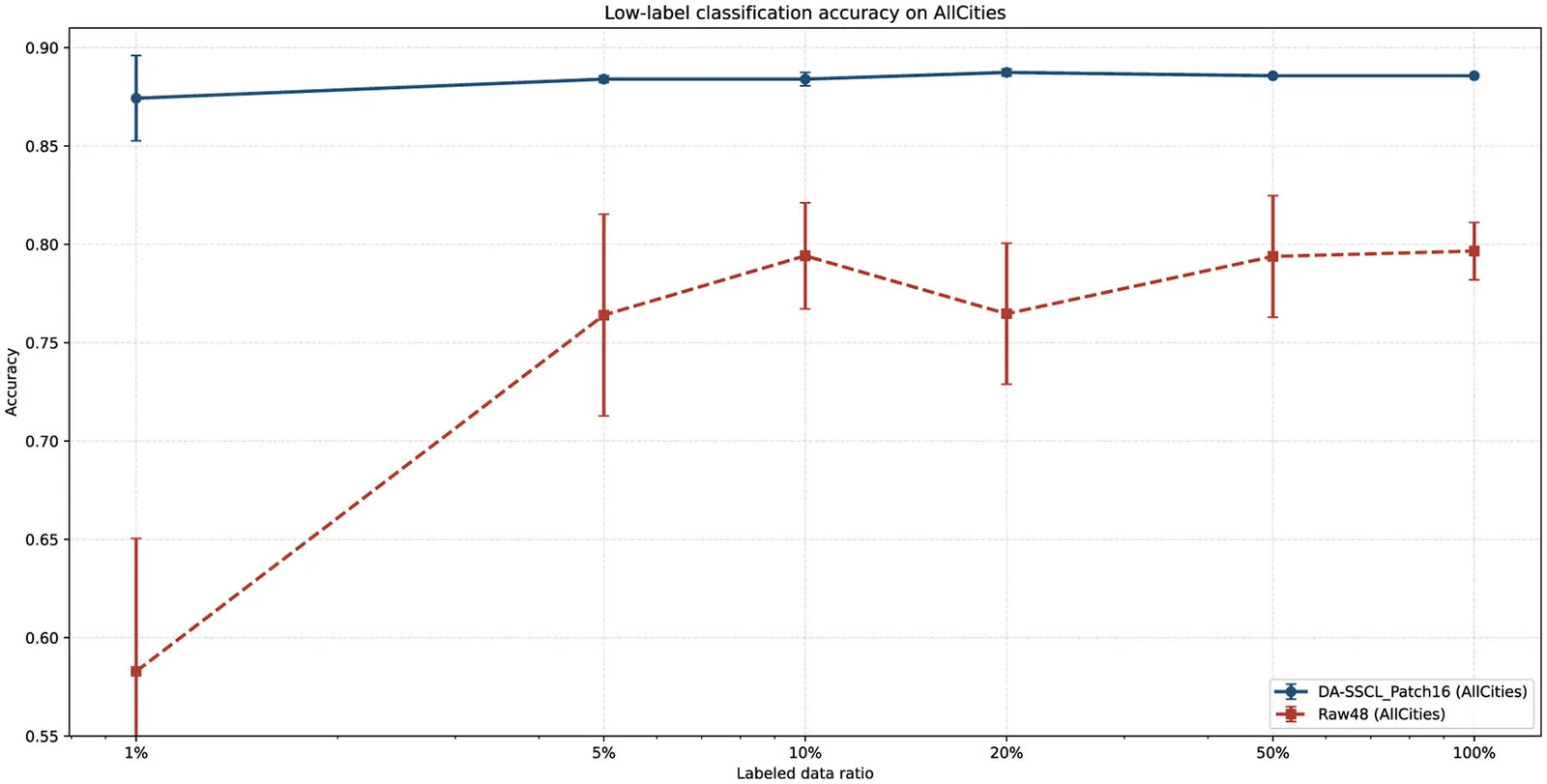
Classification accuracy under limited label scenarios (DA-SSCL_Patch16 vs. Raw48). The curves show the accuracy trend as the labeled data ratio increases from 1 to 100%.

Figure 3 shows that DA-SSCL reaches 88.4% accuracy with only 10% labeled data, which is already close to the fully supervised setting. Even when only 1% labeled data are used, the accuracy remains competitive. This indicates that much of the discriminative structure of user behavior is captured during self-supervised pretraining, reducing the dependence on large-scale downstream annotation. For practical AMI applications, this label efficiency is important because obtaining high-quality annotations for a large number of users is usually expensive and labor-intensive.

Table 11 shows that this label-efficiency advantage is not limited to the comparison with raw features. At the 10% labeling ratio, DA-SSCL not only outperforms generic foundation models such as MOMENT16 and TSDE16 by a clear margin, but also maintains an advantage over the specialized Series2Vec16 baseline. The margin over Series2Vec16 is small but consistent, indicating that benchmark-grounded invariance design improves not only unsupervised clustering but also representation separability under scarce labels. This supports the central claim of the paper: physically grounded pretraining objectives produce embeddings that are more reusable for downstream monitoring tasks. We report the main stochastic representation, augmentation, transfer, and hyperparameter experiments as mean ± std over three random seeds; the 10% semi-supervised table is retained as a focused label-efficiency comparison under the same support-ratio protocol.

**Table 11:** Semi-supervised comparison on AllCities at 10% labels.

Method	Accuracy	Macro F1
Raw48	0.794	0.800
MOMENT16 (Goswami et al., 2024)	0.697	0.673
TSDE16 (Senane et al., 2024)	0.797	0.795
Series2Vec16 (Foumani et al., 2024)	0.870	0.874
**DA-SSCL_Patch16**	**0.884**	**0.879**

Bold values indicate the best performance among the compared methods; the proposed method is also shown in bold for identification.

## 6 Discussion

### 6.1 Interpretability and feature space geometry

#### 6.1.1 Temporal importance and generative factors

While the semi-supervised results confirm the downstream discriminative power of DA-SSCL, it is also necessary to clarify *what* the encoder responds to and how these representations capture the underlying generative factors of the time-series data. PatchMLP is not an attention-based model, so the visualization in this section should not be interpreted as learned attention weights. Instead, we compute *post-hoc* temporal importance by occlusion sensitivity: each temporal position is masked in turn, the resulting change in the normalized embedding is measured, and the scores are averaged over samples and normalized across the 24-h input sequence.

**Figure 4:**
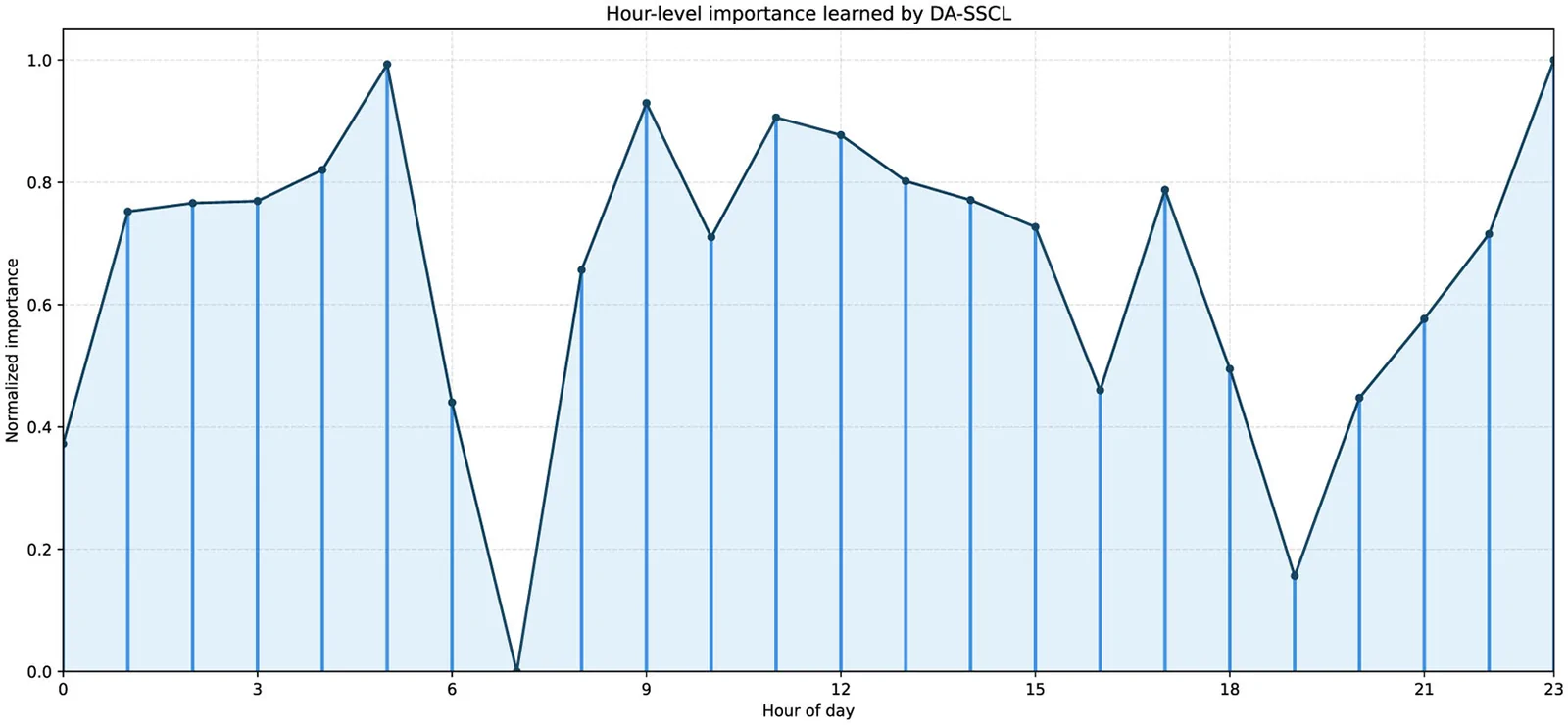
Temporal importance profile on the input sequence. The *post-hoc* occlusion-sensitivity distribution indicates that intervals with high behavioral variability (e.g., morning and evening structural shifts) contribute more strongly to the learned embedding than stationary background periods.

Figure 4 indicates that masking the 7:00 a.m.–12:00 p.m. window and the evening active period produces larger embedding changes than masking more stationary periods. In the context of cyber-physical systems, these intervals correspond to periods of high behavioral entropy and policy-driven structural shifts (e.g., EV charging onset, TOU pricing response). This temporal-importance profile suggests that the benchmark-grounded contrastive objective helps the encoder preserve salient generative factors of the temporal data, rather than relying only on stationary background patterns.

#### 6.1.2 Embedding space visualization

To further evaluate the quality of the learned representations, we visualize the geometry of the high-dimensional feature space. A robust time-series SSL model should map structurally similar sequences to adjacent regions while maintaining sufficient margin between distinct behavioral modes, even under severe domain shift.

**Figure 5:**
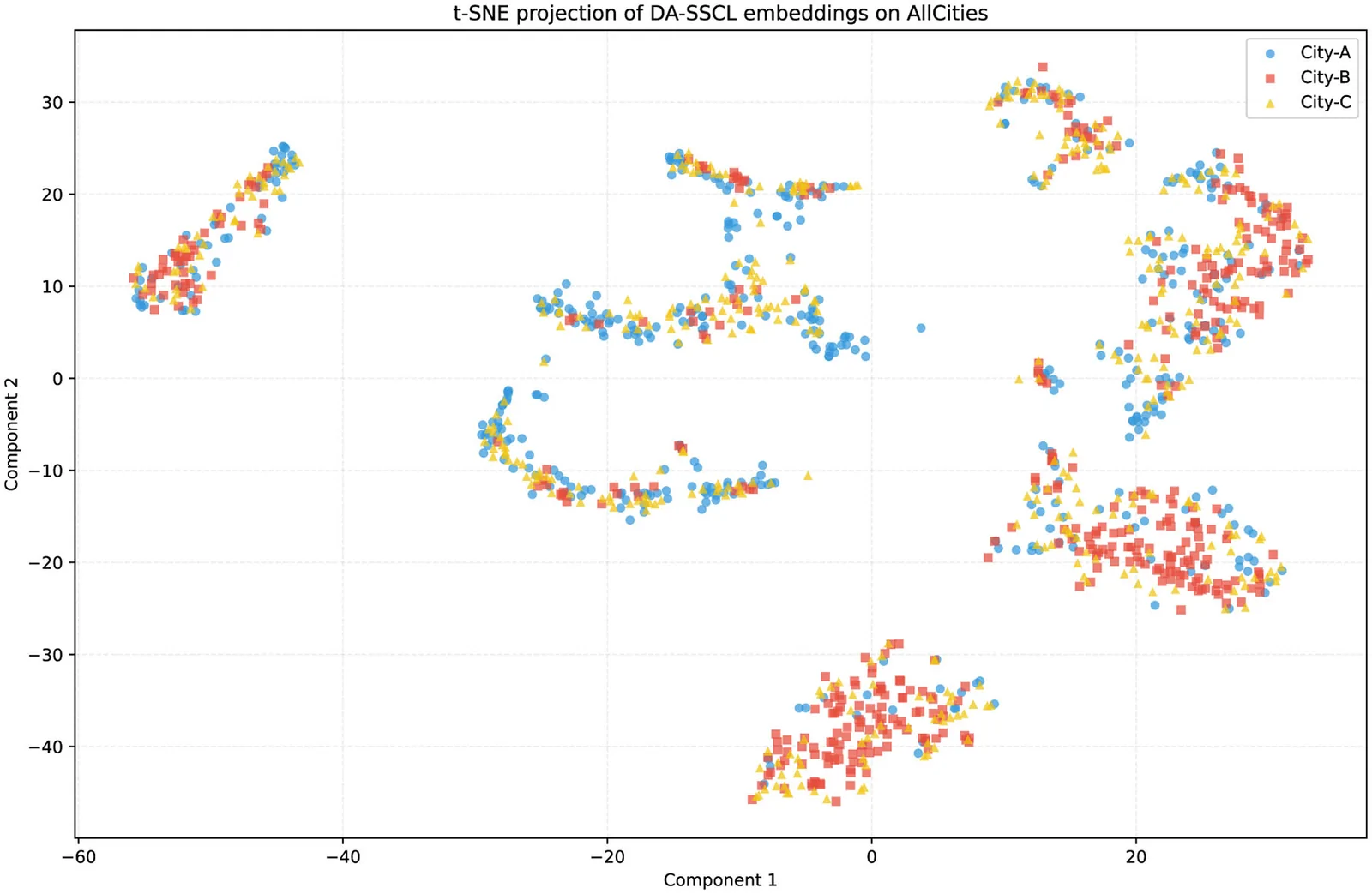
t-SNE projection of the learned feature space across heterogeneous domains (AllCities benchmark). The projection qualitatively illustrates that DA-SSCL organizes distinct behavioral modes into compact and partially connected regions; quantitative evidence is reported in the ARI, NMI, silhouette, and transfer results.

Figure 5 provides a qualitative view that is consistent with the quantitative results in Tables 7, 10. The embedding space shows compact regions associated with distinct temporal structures. The t-SNE projection is used only as an explanatory visualization; the quantitative evidence for cluster alignment, compactness, and cross-domain utility is provided by ARI, NMI, silhouette coefficient, and the transfer-control metrics. The visualization also suggests that district-specific structures are not completely isolated in the learned space, which is consistent with the observed cross-district transfer performance. We therefore interpret Figure 5 as supporting qualitative evidence, rather than as standalone quantitative evidence, that DA-SSCL learns representations suitable for transfer learning within the benchmarked utility districts.

### 6.2 Implications for AI in cyber-physical systems

The empirical results of DA-SSCL offer broader implications for representation learning in industrial AI. First, the framework shows that incorporating domain physics into the self-supervised pretext task—rather than relying solely on larger models or generic augmentations—can improve label efficiency. The ability to achieve 88.4% accuracy with only 10% labels provides a practical pathway for deploying deep learning in domains where expert annotation is a primary bottleneck.

Second, the transfer learning evaluation shows that a lightweight architecture (PatchMLP), when trained with a physically-grounded contrastive objective, can generalize across the evaluated district-level spatial shifts. The paradigm of source-domain pretraining followed by unlabeled target-domain adaptation (yielding an 89.7% target accuracy) provides a practical approach for continuous learning in non-stationary cyber-physical environments. Finally, by open-sourcing the WCA-Bench with expert-verified structural evolution events, we provide the AI community with a benchmark for evaluating future advances in time-series out-of-distribution (OOD) generalization and anomaly detection.

### 6.3 Limitations and future directions

While DA-SSCL demonstrates strong performance in representation quality and transferability, several avenues for future research remain. First, the current framework relies on a predefined augmentation library derived from domain expertise. When encountering entirely novel OOD phenomena (e.g., unprecedented grid faults or extreme weather anomalies), these fixed priors may be insufficient. Future work should explore automated, data-driven augmentation search strategies or generative adversarial perturbations to dynamically expand the invariance space. Second, while our user-level split prevents the same user from appearing in both training and test partitions, daily AMI profiles are naturally temporally correlated; therefore, temporal generalization (e.g., training on historical years and testing on future years with drifted distributions) remains to be fully explored with chronological splits. Third, the present interpretability analysis focuses on temporal importance and embedding geometry; more systematic comparisons with attribution methods such as SHAP or Integrated Gradients, together with quantitative manifold scores beyond ARI/NMI/SC, would further strengthen explanation quality. Finally, extending this framework to handle multimodal cyber-physical streams (e.g., jointly modeling load, voltage, and frequency topologies) via cross-modal contrastive learning represents a promising frontier for holistic system state representation.

An additional practical limitation concerns privacy and cybersecurity. Although the released benchmark is anonymized and aggregated at the daily-profile level, learned representations from AMI data may still encode sensitive behavioral information if deployed without governance controls. Future work should therefore investigate privacy-preserving representation learning, secure model update pipelines, access-controlled embedding storage, and federated or differentially private pretraining schemes for utility-facing deployment.

## 7 Conclusion

In this paper, we address time-series representation learning in complex, non-stationary cyber-physical systems. We introduce the WCA-Bench, a real-world benchmark comprising 713,700 temporal profiles and 506 expert-verified structural evolution events. To tackle the limitations of generic SSL methods on such data, we propose DA-SSCL, a domain-aware contrastive learning framework that translates physical sensing phenomena into representation learning constraints.

The experiments support the effectiveness of this approach: DA-SSCL achieves an unsupervised clustering ARI of 0.699, reaches 88.4% semi-supervised classification accuracy with only 10% labels, and obtains strong cross-district transfer performance (89.7% target accuracy and 0.8967 Macro-F1). These findings indicate that grounding self-supervised objectives in domain physics can yield representations that are robust, interpretable, and transferable within the evaluated AMI benchmark. By releasing both the framework and the WCA-Bench, we aim to support future research in robust time-series representation learning, domain adaptation, and label-efficient AI for industrial applications.

## Data Availability

The WCA-Bench dataset supporting the conclusions of this article is available from the corresponding author upon reasonable request. Requests to access the dataset should be directed to Yumin Yao at yaoyumin@csu.edu.cn.
